# Prognostic value of triglyceride-glucose index in patients with chronic coronary syndrome undergoing percutaneous coronary intervention

**DOI:** 10.1186/s12933-023-02060-7

**Published:** 2023-11-28

**Authors:** Shiyi Tao, Lintong Yu, Jun Li, Zicong Xie, Li Huang, Deshuang Yang, Yuqing Tan, Wenjie Zhang, Xuanchun Huang, Tiantian Xue

**Affiliations:** 1 Department of Cardiology, Guang’anmen Hospital, China Academy of Chinese Medical Sciences, Beijing, China; 2 Graduate School, Beijing University of Chinese Medicine, Beijing, China; 3 Department of Integrative Cardiology, China-Japan Friendship Hospital, Beijing, China

**Keywords:** Triglyceride-glucose index, Coronary artery Disease, Chronic coronary syndrome, Percutaneous coronary intervention, Major adverse cardiovascular events, Insulin resistance

## Abstract

**Background:**

The triglyceride-glucose (TyG) index has been proposed as a reliable surrogate marker of insulin resistance and an independent predictor of major adverse cardiovascular events (MACEs). Several recent studies have shown the relationship between the TyG index and cardiovascular outcomes; however, the role of the TyG index in chronic coronary syndrome (CCS) progression has not been extensively assessed especially in population after revascularization. This study aimed to investigate the prognostic value of the TyG index in predicting MACEs in CCS patients undergoing percutaneous coronary intervention (PCI).

**Methods:**

The data for the study were taken from the Hospital Information System database in China-Japan Friendship Hospital over the period 2019–2021. Eligible participants were divided into groups according to the TyG index tertiles. The Boruta algorithm was performed for feature selection. Multivariate Cox proportional hazards models and restricted cubic spline (RCS) analysis were applied to examine the dose–response relationship between the TyG index and endpoint, and the results were expressed with hazard ratio (HR) and 95% confidence interval (CI) values. The area under the receiver operating characteristic (ROC) curve (AUC), decision curve analysis (DCA), and clinical impact curve (CIC) were plotted to comprehensively evaluate the predictive accuracy and clinical value of the model. The goodness-of-fit of models was evaluated using the calibration curve and χ^2^ likelihood ratio test.

**Results:**

After applying inclusion and exclusion criteria, 1353 patients with CCS undergoing PCI were enrolled in the study. After adjusting for all confounders, we found that those with the highest TyG index had a 59.5% increased risk of MACEs over the 1-year follow-up (HR 1.595, 95% *CI* 1.370 ~ 1.855). Using the lowest TyG index tertile as the reference (T1), the fully adjusted HRs (95% *CI*s) for endpoints was 1.343 (1.054 ~ 1.711) in the middle (T2) and 2.297 (1.842 ~ 2.864) in highest tertile (T3) (*P* for trend < 0.001). The TyG index had an excellent predictive performance according to the results of AUC 0.810 (0.786, 0.834) and *χ*^*2*^ likelihood ratio test (*χ*^*2*^ = 7.474, *P* = 0.486). DCA and CIC analysis also suggested a good overall net benefit and clinical impact of the multivariate model. The results in the subgroup analysis were consistent with the main analyses. RCS model demonstrated that the TyG index was nonlinearly associated with the risk of MACEs within one year (*P* for nonlinear < 0.001).

**Conclusion:**

The elevated TyG index is associated with an increased risk of cardiovascular events and predicts future MACEs in patients with CCS undergoing PCI independently of known cardiovascular risk factors, indicating that the TyG index may be a potential marker for risk stratification and prognosis in CCS patients undergoing PCI.

**Supplementary Information:**

The online version contains supplementary material available at 10.1186/s12933-023-02060-7.

## Introduction

Coronary artery disease (CAD) has affected 244.11 million individuals worldwide and is the leading cause of death, constituting an increasing public health burden worldwide [1, 2]. Chronic coronary syndrome (CCS), previously referred to as stable CAD, characterized by the interruption of the coronary artery intimal layer and intramural hematoma, causing vessel compression, and typically presenting as an acute coronary syndrome (ACS) [3]. CCS contributes to the major population of CAD and encompasses patients with or without previous ACS or revascularization [4].

Observational studies indicate that most conservatively managed patients recover without further intervention [5–7]. However, in patients with ongoing ischemia, vessel occlusion, or patient instability, selective revascularization may be necessary [4, 8]. Percutaneous coronary intervention (PCI) with drug-eluting stent (DES) is one of the most conventional revascularization strategies performed in CCS patients. Although the management of PCI-DES in CCS patients has made great advances over the past few decades, the incidence of major adverse cardiovascular events (MACEs) such as repeat revascularization and in-stent restenosis may still exceeds 25% at 5-year follow-up [9, 10]. Therefore, it is critically important to identify high-risk patients of suffering from future MACEs so that intense management can be offered. The identification of rapidly available and reliable markers may have great clinical significance in optimizing the risk stratification of recurrent cardiovascular risk.

Insulin resistance (IR) is a clinical state of impaired insulin sensitivity which may lead to cardiometabolic alterations such as hyperglycemia, dyslipidemia, and hypertension [11, 12]. Previous evidence indicated that IR was a strong predictor of increased cardiovascular morbidity and mortality [13–15]. The triglyceride-glucose (TyG) index, combined with fasting glucose and triglycerides, has been proposed as a reliable surrogate marker for IR [16–18]. Growing studies demonstrated that the TyG index may independently predict adverse cardiovascular outcomes among CAD cohorts with different clinical manifestations [19–21]. However, there are limited clinical studies assessing the prognostic value of the TyG index in CCS patients undergoing PCI.

This study aimed to examine the association between the TyG index and the risk of MACEs and to determine the prognostic value of the TyG index in CCS patients undergoing PCI through accessible real-world data. The findings of this study may help identify high-risk individuals and develop clinical strategies to improve outcomes in these specific populations at the earliest, and provide crucial new insights into the research of TyG in predicting outcomes in the field of CAD.

## Materials and methods

### Study design and participants

**Fig. 1:**
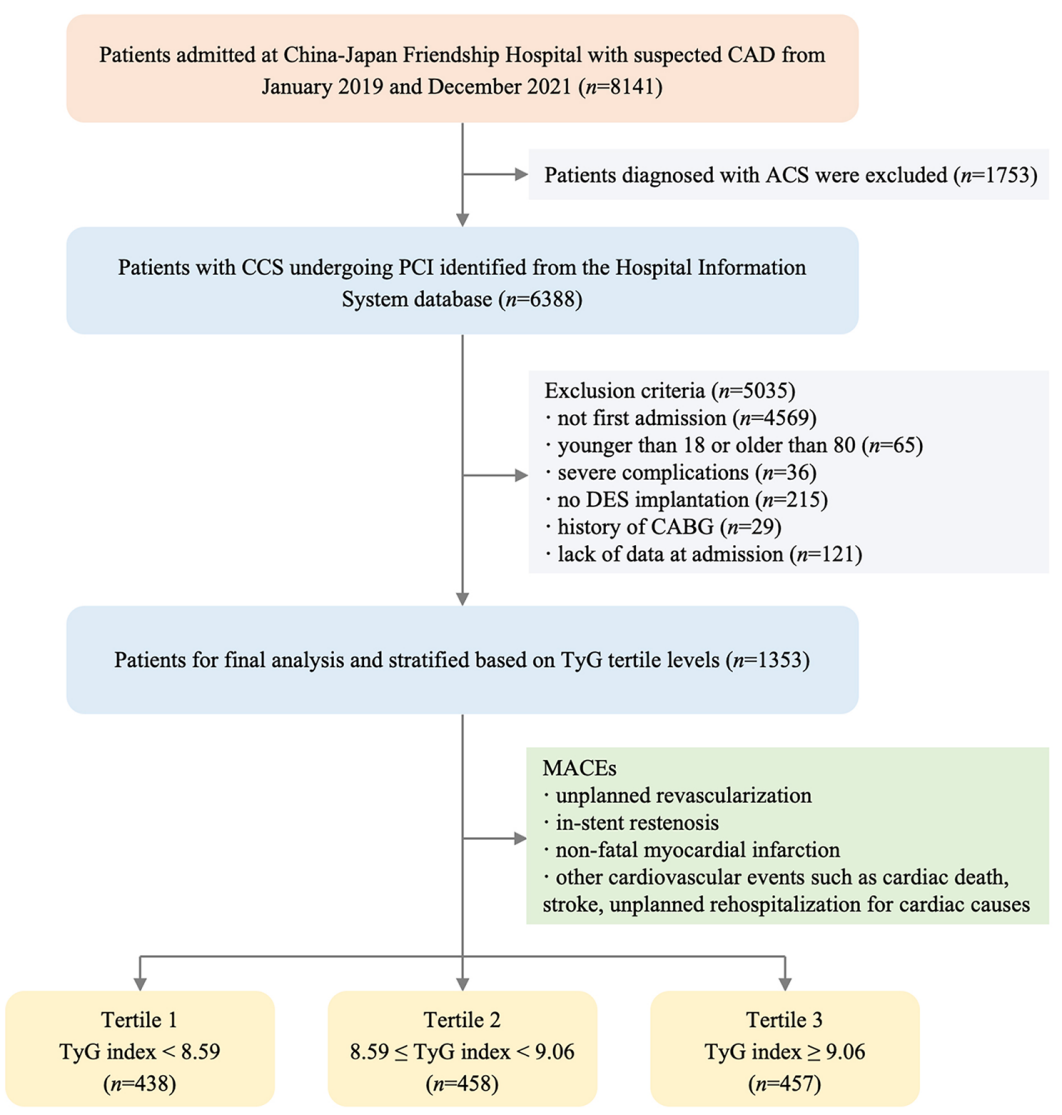
Flow diagram of patient selection. TyG, triglyceride-glucose; MACE, major adverse cardiovascular event; CAD, coronary artery disease; ACS, acute coronary syndrome; CCS, chronic coronary syndrome; PCI, percutaneous coronary intervention; DES, drug-eluting stent; CABG, coronary artery bypass grafting

This was a single-center, retrospective, observational cohort study. Admission data of consecutive CCS patients undergoing PCI were collected and assessed from the Hospital Information System database in China-Japan Friendship Hospital between January 2019 and December 2021. The definition of CCS complied with the current guideline of the European Society of Cardiology [3]. Among the 8141 patients, 6388 patients were excluded based on the study exclusion criteria, which included (1) younger than 18 or older than 80; (2) not first admission or lack of data at admission; (3) no DES implantation; (4) severe complications such as advanced cancer, severe hepatic and renal dysfunction, severe hematological and endocrine system diseases; and (5) history of coronary artery bypass grafting. Eventually, 1353 eligible patients were enrolled in this study and classified into three groups according to the TyG index tertiles (Fig. 1). This retrospective study was performed in line with the Declaration of Helsinki. Because of the retrospective design of this study, the need for informed consent was waived by the institutional review board, and information related to patient identity was concealed.

### Data collection and definitions

Clinical data, including demographic characteristics, the clinical history, laboratory indicators, echocardiography and peripheral arterial disease features, and number of coronary lesions and stent implantation, were collected using a standardized questionnaire by trained clinicians who were blinded to the purpose of the study. Demographic characteristics included weight, height [to calculate body mass index (BMI)], age, gender, baseline systolic blood pressure (SBP) and diastolic blood pressure (DBP), heart rate (HR), smoking history and drinking history. Clinical history included established diabetes mellitus, hypertension, stroke, old myocardial infarction (OMI), dyslipidemia, and history of cardiovascular diseases (CVDs). Laboratory tests consisting of neutrophil (Neu), lymphocyte (Lym), platelets (PLT), hemoglobin (Hb), alanine aminotransferase (ALT), aspartate aminotransferase (AST), total cholesterol (TC), triglyceride (TG), low density lipoprotein cholesterol (LDL-C), high density lipoprotein cholesterol (HDL-C), lipoprotein (a) [Lp (a)], homocysteine (HCY), hypersensitive C-reactive protein (Hs-CRP), serum creatinine (Scr), fasting blood glucose (FBG) and glycated hemoglobin A1c (HbA1c) were performed under standardized instructions and assaying system. All blood samples were collected after overnight fasting before coronary angiography. Echocardiography features consisting of left atrial diameter (LAD), left ventricular end-diastolic diameter (LVDd), interventricular septal thickness (IVST), left ventricular posterior wall thickness (PWT), and left ventricular ejection fraction (LVEF) were analyzed and recorded by two independent echocardiographers. The angiographic data was obtained from the cardiac catheterization laboratory records. Peripheral arterial disease indicators included brachial-ankle pulse wave velocity (baPWV), ankle-brachial index (ABI), and brachial artery flow-mediated vasodilatation (FMD) value. The TyG index was calculated as Ln [fasting TG (mg/dL) × FBG (mg/dL)/2] [17].

### Feature selection

Boruta algorithm, characterized by an extension of the random forest algorithm and the creation of “shadow features” by shuffling the real features, was utilized to identify the most critical features related to the risk of MACEs and to establish the radiomics signatures. A replicated feature called the shadow feature was created by Boruta-based method from the original dataset to compare the Z-score between the genuine features and the shadow feature generated by the random forest classifier in each iteration of the model development. The Z-value of each feature is evaluated based on its importance in the random forest model, and the maximum Z-value of the shadow features is recorded. A real feature is considered important if its Z-value is greater than the maximum Z-value of the shadow features; otherwise, it was eliminated [22].

### Follow-up and endpoints

Clinical follow-up was carried out by skilled clinicians in outpatient or telephone contact at the time points of one year, and standard computerized case report forms were filled out. The endpoint events were independently categorized by three cardiovascular specialists who were not aware of the baseline information. When there were disagreements regarding event identification, the three experts came to a decision together after talking.

The primary endpoint of this clinical trial was defined as a compound endpoint of the first occurrence of total MACEs within one-year follow up. The total MACEs was defined as follows: (1) unplanned revascularization, which means that the patient underwent revascularization again due to unexpected internal cardiac causes; (2) in-stent restenosis, which was defined as 50% or more of the target vessel stenosis within 5 mm from the edge of the stent or both ends of the stent after percutaneous coronary intervention as shown by coronary angiography; (3) non-fatal myocardial infarction, referring to myocardial necrosis but no death, accompanied by ischemia symptoms, abnormal myocardial markers, ST segment changes or pathological Q wave changes; (4) other cardiovascular events such as cardiac death, stroke, unplanned rehospitalization for cardiac causes.

### Statistical analysis

Continuous variables were expressed as mean ± standard deviation (SD) and median [interquartile range (IQR)] for those with normal and skewed distributions, respectively. Categorical variables were expressed as number (percentage). All participants were stratified into three groups: T1 (TyG index < 8.59, *n* = 438), T2 (8.59 ≤ TyG index < 9.06, *n* = 458), and T3 (TyG index ≥ 9.06, *n* = 457) in accordance with the TyG index tertiles. Continuous data were compared using one-way analysis of variance or the Kruskal-Wallis test among the three groups according to the presence or absence of normal distribution, and the chi-squared test or Fisher’s exact test was performed for categorical variables.

The Boruta-based feature selection method was performed to determine the potential markers associated with endpoints. The cumulative incidence of endpoints was described by the Kaplan-Meier method and compared between groups using the log-rank test. Multivariate Cox proportional hazards models were applied to test the associations of the TyG index with endpoints, and the results were expressed as hazard ratio (HR) and 95% confidence interval (CI) values. Risk factors that were statistically significant in the univariate analysis (*P* < 0.05), screened by Boruta algorithm, or clinically significant were selected as candidates for the multivariate model. Besides the Model 1 without any other adjustments for confounding factors, two other models were fitted. In Model 2, age, gender, BMI, SBP, and DBP were modified. Model 3 was a completely adjusted model that took feature selection results and clinical experience adjustments into account. The linear trends across TyG tertiles were evaluated by a median value within each tertile as a continuous variable. Additionally, multivariate restricted cubic spline (RCS) analysis was used to assess any potential nonlinear relationships between the TyG index and endpoints. The area under the receiver operating characteristic (ROC) curve (AUC), decision curve analysis (DCA), and clinical impact curve (CIC) were plotted to comprehensively evaluate the predictive accuracy and clinical value of the model. The goodness-of-fit of models was evaluated using the calibration curve and *χ*^*2*^ likelihood ratio test. Furthermore, we performed subgroup and interaction analyses based on age(< 65 years or ≥ 65 years), gender (male or female), BMI (< 24 kg/m^2^ or ≥ 24 kg/m^2^), diabetes (yes or no), hypertension (yes or no), familial CVDs (yes or no), LDL-C (yes or no), multi-vessel disease (yes or no), and multi-DES implantation (yes or no), to identify whether the relationships between the TyG index and endpoints varied according to the status of the potential covariates.

All statistical analyses were performed using IBM-SPSS (version 26.0, Chicago, IL, USA) and R (version 4.1.2, Vienna, Austria). A two-sides *P* value of less than 0.05 was considered to indicate statistical significance.

## Results

### Baseline characteristics

Overall, a total of 1353 CCS patients undergoing PCI from the Hospital Information System database were included in the analysis with a median TyG index of 8.83 (8.47, 9.18). The median follow-up time was 1.7 years. Among the 1353 eligible patients, 542 (40.06%) suffered from MACEs within one year while 811 did not. The baseline characteristics of the study population are summarized in Table 1 and Additional file 1: Table S1.

The average age of the participants was 62 years, and 72.36% of the participants were male. Patients with higher baseline TyG index had a greater prevalence of MACEs and comorbidities (including diabetes, hypertension, stroke, and dyslipidemia) and higher ratios of patients with familial CVDs and a history of multi-DES implantation. They also had higher BMI, SBP, HR, Neu, Lym, PLT, ALT, TC, TG, LDL-C, HCY, Hs-CRP, FBG, and HbA1c (all *P* < 0.05). Furthermore, the highest TyG index tertile were more likely to have higher baPWV, lower ABI and FMD, and higher LAD, LVDd, IVST, and PWT (all *P* < 0.05), indicating a worse peripheral artery condition and poorer cardiac performance. Additionally, the proportions of individuals with the use of antiplatelet medication such as aspirin, nitrates were significantly lower in this group, as well as the HDL-C level. Collinearity diagnostics showed that no potentially significant collinearity was observed among variables (Additional file 1: Table S2).

**Table 1:** Baseline characteristics of the study population according to the TyG index tertiles

Variables	Total (*n* = 1353)	T1 (*n* = 438)	T2 (*n* = 458)	T3 (*n* = 457)	*P* value
TyG index	8.83 (8.47, 9.18)	8.3 (8.07, 8.46)	8.82 (8.72, 8.93)	9.32 (9.17, 9.58)	< 0.001
MACEs (n, %)	542 (40.06)	129 (29.45)	148 (32.31)	265 (57.99)	< 0.001
Demographics					
Male (n, %)	979 (72.36)	328 (74.89)	325 (70.96)	326 (71.33)	0.352
Age (years)	64 (57, 71)	63 (56, 71)	64 (57, 70)	67 (58, 74)	< 0.001
BMI (kg/m^2^)	24.97 (23.29, 27.18)	24.37 (22.56, 26.42)	24.79 (23.24, 27.43)	25.61 (23.88, 27.66)	< 0.001
SBP (mmHg)	134 (123, 143)	134 (121, 143)	134 (122, 140)	134 (127, 146)	0.008
DBP (mmHg)	78 (71, 84)	78 (70, 84)	78 (71, 82)	78 (73, 85)	0.194
HR (bpm)	74 (68, 79)	73 (66, 78)	74 (68, 76)	74 (70, 80)	< 0.001
Case history (n, (%)					
Smoking	599 (44.27)	180 (41.1)	201 (43.89)	218 (47.7)	0.135
Drinking	300 (22.17)	94 (21.46)	95 (20.74)	111 (24.29)	0.395
Diabetes	612 (45.23)	144 (32.88)	207 (45.2)	261 (57.11)	< 0.001
Hypertension	1026 (75.83)	305 (69.63)	361 (78.82)	360 (78.77)	0.001
Stroke	233 (17.22)	76 (17.35)	59 (12.88)	98 (21.44)	0.003
OMI	238 (17.59)	77 (17.58)	77 (16.81)	84 (18.38)	0.824
Dyslipidemia	889 (65.71)	280 (63.93)	281 (61.35)	328 (71.77)	0.003
Familial CVDs	366 (27.05)	101 (23.06)	122 (26.64)	143 (31.29)	0.021
Coronary lesions (n, %)					
One-vessel disease	963 (71.18)	319 (72.83)	329 (71.83)	315 (68.93)	0.405
Two-vessel disease	299 (22.1)	92 (21)	100 (21.83)	107 (23.41)	0.676
Multi-vessel disease	91 (6.73)	27 (6.16)	29 (6.33)	35 (7.66)	0.617
Number of stents (n, %)					
One-DES implantation	625 (46.19)	211 (48.17)	221 (48.25)	193 (42.23)	0.113
Two-DES implantation	426 (31.49)	127 (29)	161 (35.15)	138 (30.2)	0.107
Multi-DES implantation	302 (22.32)	100 (22.83)	76 (16.59)	126 (27.57)	< 0.001
Cardiovascular medications (n, %)					
Aspirin	1337 (98.82)	435 (99.32)	456 (99.56)	446 (97.59)	0.011
Clopidogrel/Ticagrelor	1348 (99.63)	438 (100)	456 (99.56)	454 (99.34)	0.259
Statins	1305 (96.45)	429 (97.95)	441 (96.29)	435 (95.19)	0.081
ACEI/ARB	671 (49.59)	210 (47.95)	238 (51.97)	223 (48.8)	0.444
*β*-blockers	950 (70.21)	302 (68.95)	324 (70.74)	324 (70.9)	0.780
CCB	440 (32.52)	128 (29.22)	147 (32.1)	165 (36.11)	0.087
Nitrates	361 (26.68)	121 (27.63)	140 (30.57)	100 (21.88)	0.010
Diuretics	278 (20.55)	93 (21.23)	84 (18.34)	101 (22.1)	0.338
Laboratory measurements					
Neu (10^9^/L)	4.36 (3.4, 4.99)	4.12 (3.08, 4.74)	4.36 (3.54, 4.87)	4.36 (3.64, 5.23)	< 0.001
Lym (10^9^/L)	1.81 (1.43, 2.08)	1.71 (1.36, 2)	1.81 (1.46, 2.08)	1.81 (1.49, 2.18)	< 0.001
PLT (10^9^/L)	209 (174, 238)	207 (164, 232)	209 (179, 231)	209 (179, 248)	0.005
Hb (g/L)	134 (126, 144)	134 (124, 143)	134 (128, 145)	134 (126, 146)	0.099
ALT (U/L)	21 (14, 30)	19 (14, 26)	22 (15, 30)	23 (15, 35)	< 0.001
AST (U/L)	19 (16, 25)	19 (16, 24)	19 (16, 25)	19 (15, 26)	0.647
TC (mmol/L)	3.83 (3.21, 4.71)	3.51 (2.89, 4.08)	3.77 (3.2, 4.67)	4.42 (3.58, 5.25)	< 0.001
TG (mmol/L)	1.4 (1.04, 1.9)	0.89 (0.76, 1.06)	1.43 (1.22, 1.64)	2.13 (1.8, 2.69)	< 0.001
LDL-C (mmol/L)	2.29 (1.86, 2.88)	2.09 (1.62, 2.5)	2.27 (1.87, 2.9)	2.63 (2.08, 3.24)	< 0.001
HDL-C (mmol/L)	1.01 (0.86, 1.17)	1.05 (0.89, 1.25)	1 (0.87, 1.14)	0.97 (0.82, 1.13)	< 0.001
Lp (a) (mg/L)	159.75 (55.76, 227.47)	150.65 (52.9, 232.88)	171.42 (55.76, 246.13)	168.67 (58.83, 219.74)	0.768
HCY (*µ*mol/L)	13.65 (11.34, 17.31)	13.39 (11.06, 16.54)	13.37 (11.06, 17.27)	14.18 (11.76, 18.48)	0.002
Hs-CRP (mg/L)	2.17 (0.93, 4.81)	1.71 (0.8, 4.81)	1.99 (0.84, 4.81)	2.74 (1.25, 4.81)	< 0.001
Scr (*µ*mol/L)	74 (62.7, 84.7)	74.1 (64.3, 83.9)	74 (62.9, 84.8)	73.9 (61.5, 87.1)	0.946
FBG (mmol/L)	6.11 (5.22, 6.54)	5.32 (4.85, 6.21)	6.11 (5.31, 6.29)	6.43 (6, 7.74)	< 0.001
HbA1c (%)	6.1 (5.7, 7.1)	5.9 (5.6, 6.4)	6.2 (5.8, 6.9)	6.6 (5.8, 7.9)	< 0.001
PAD indicators					
baPWV (m/s)	17.04 (15.32, 21.68)	16.21 (15.21, 20.82)	16.8 (15.12, 21.12)	19.28 (15.66, 22.66)	< 0.001
ABI	1.09 (0.96, 1.18)	1.12 (0.98, 1.18)	1.11 (0.99, 1.18)	1.03 (0.9, 1.16)	< 0.001
FMD (%)	7 (6.1, 8.4)	7.1 (6.3, 8.5)	7.2 (6.2, 8.5)	6.6 (6, 8.2)	< 0.001
Echocardiography					
LAD (mm)	37 (35, 40)	36 (34, 39.25)	37.69 (36, 39)	38 (35, 40)	< 0.001
LVEF (%)	65 (60, 70)	66 (61, 70)	66 (60, 70)	65 (59, 70)	0.163
LVDd (mm)	50 (47, 53)	50 (47, 53)	50 (47, 53)	51 (47, 54)	0.031
IVST (mm)	10 (9, 11)	10 (9, 11)	10 (9, 11)	10 (10, 11)	< 0.001
PWT (mm)	9 (8, 10)	9 (8, 9.6)	9 (8, 10)	9 (8, 10)	< 0.001

TyG, triglyceride-glucose; T1, tertile 1; T2, tertile 2; T3, tertile 3; MACE, major adverse cardiovascular event; BMI, body mass index; SBP, systolic blood pressure; DBP, diastolic blood pressure; HR, heart rate; OMI, old myocardial infarction; CVD, cardiovascular disease; DES, drug-eluting stent; ACEI, angiotensin converting enzyme inhibitor; ARB, angiotensin receptor blocker; CCB, calcium channel blockers; Neu, neutrophil; Lym, lymphocyte; PLT, platelets; Hb, hemoglobin; ALT, alanine aminotransferase; AST, aspartate aminotransferase; TC, total cholesterol; TG, triglyceride; LDL-C, low-density lipoprotein cholesterol; HDL-C, high-density lipoprotein cholesterol; Lp (a), lipoprotein (a); HCY, homocysteine; Hs-CRP, hypersensitive C-reactive protein; Scr, serum creatinine; FBG, fasting blood glucose; HbA1c, glycosylated hemoglobin; PAD, peripheral artery disease; baPWV, brachial-ankle pulse wave velocity; ABI, ankle-brachial index; FMD, brachial artery flow-mediated vasodilatation; LAD, left atrial diameter; LVEF, left ventricular ejection fraction; LVDd, left ventricular end-diastolic diameter; IVST, interventricular septal thickness; PWT, left ventricular posterior wall thickness

### Feature selection

**Fig. 2:**
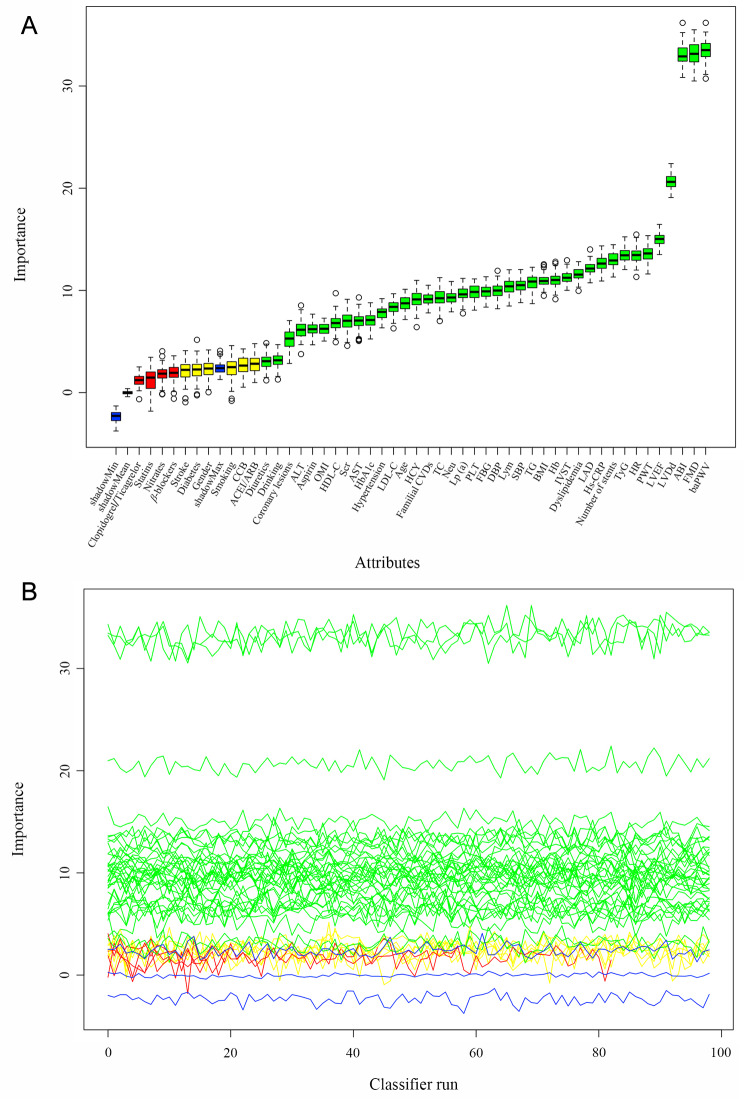
Feature selection for the potential markers associated with endpoints using the Boruta algorithm. **A**. The process of feature selection. **B**. The value evolution of Z-score in the screening process. The horizontal axis shows the name of variables and the number of iterations in Fig. 2-A and -B, respectively. While the vertical axis represents the Z-value of each variable, and the blue boxes and lines corresponds to the minimum, average, and maximum Z-scores for a shadow feature. The green boxes and lines represent confirmed variables, the yellow ones represent tentative attributes, and the red ones represent rejected variables in the model calculation. TyG, triglyceride-glucose; BMI, body mass index; SBP, systolic blood pressure; DBP, diastolic blood pressure; HR, heart rate; OMI, old myocardial infarction; CVD, cardiovascular disease; DES, drug-eluting stent; ACEI, angiotensin converting enzyme inhibitor; ARB, angiotensin receptor blocker; CCB, calcium channel blockers; Neu, neutrophil; Lym, lymphocyte; PLT, platelets; Hb, hemoglobin; ALT, alanine aminotransferase; AST, aspartate aminotransferase; TC, total cholesterol; TG, triglyceride; LDL-C, low-density lipoprotein cholesterol; HDL-C, high-density lipoprotein cholesterol; Lp (a), lipoprotein (a); HCY, homocysteine; Hs-CRP, hypersensitive C-reactive protein; Scr, serum creatinine; FBG, fasting blood glucose; HbA1c, glycosylated hemoglobin; PAD, peripheral artery disease; baPWV, brachial-ankle pulse wave velocity; ABI, ankle-brachial index; FMD, brachial artery flow-mediated vasodilatation; LAD, left atrial diameter; LVEF, left ventricular ejection fraction; LVDd, left ventricular end-diastolic diameter; IVST, interventricular septal thickness; PWT, left ventricular posterior wall thickness

Thirty-nine variables that were the most associated with the risk of MACEs were identified important after 100 iterations using the Boruta-based feature selection method, while 4 attributes were identified as unimportant and 6 tentative attributes were left (Fig. 2). Although several important characteristics, such as gender, smoking, a history of diabetes and stroke and medication situation, such as clopidogrel/ticagrelor, *β*-blockers and statins use, were disregarded because of the low Z-value in comparison to the shadow feature, they were nonetheless included in the analysis based on prior research and clinical experience. Factors were chosen for the final complete adjustment model when in the Boruta analysis, their Z-scores were higher than the shadow features or when added to the model, they had the largest matched effect (odds ratio or hazard ratio) among a group of biomarkers (max, mean and min) or they were based on previous findings and clinical constraints.

### Association between the TyG index and endpoints

**Fig. 3:**
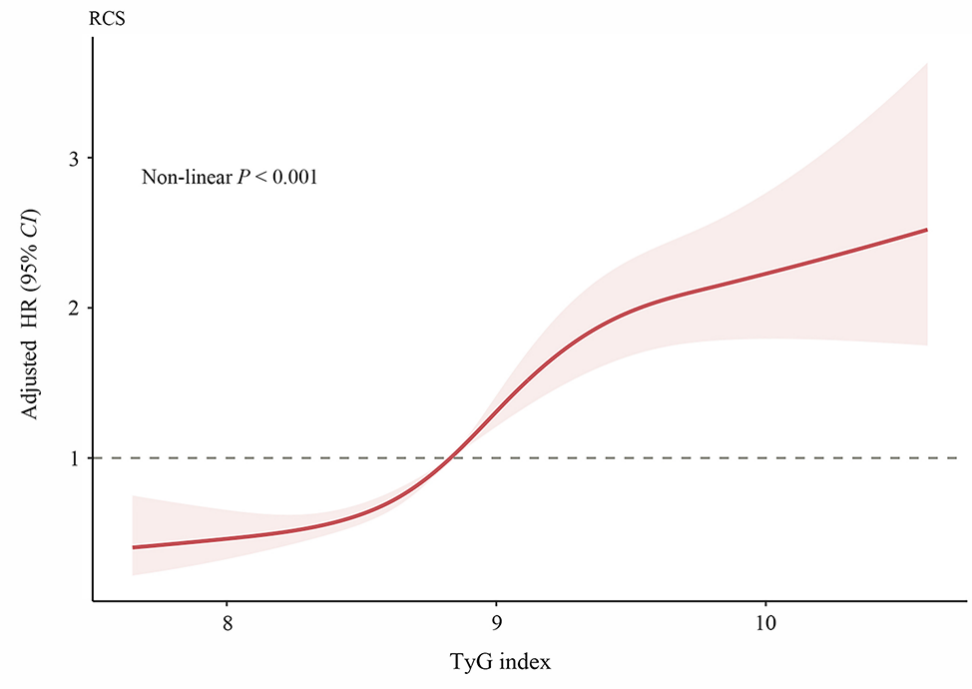
Multivariate RCS regression analysis for the nonlinear association between the TyG index and endpoints. TyG, triglyceride-glucose; HR, hazard ratio; CI, confidence interval

**Fig. 4:**
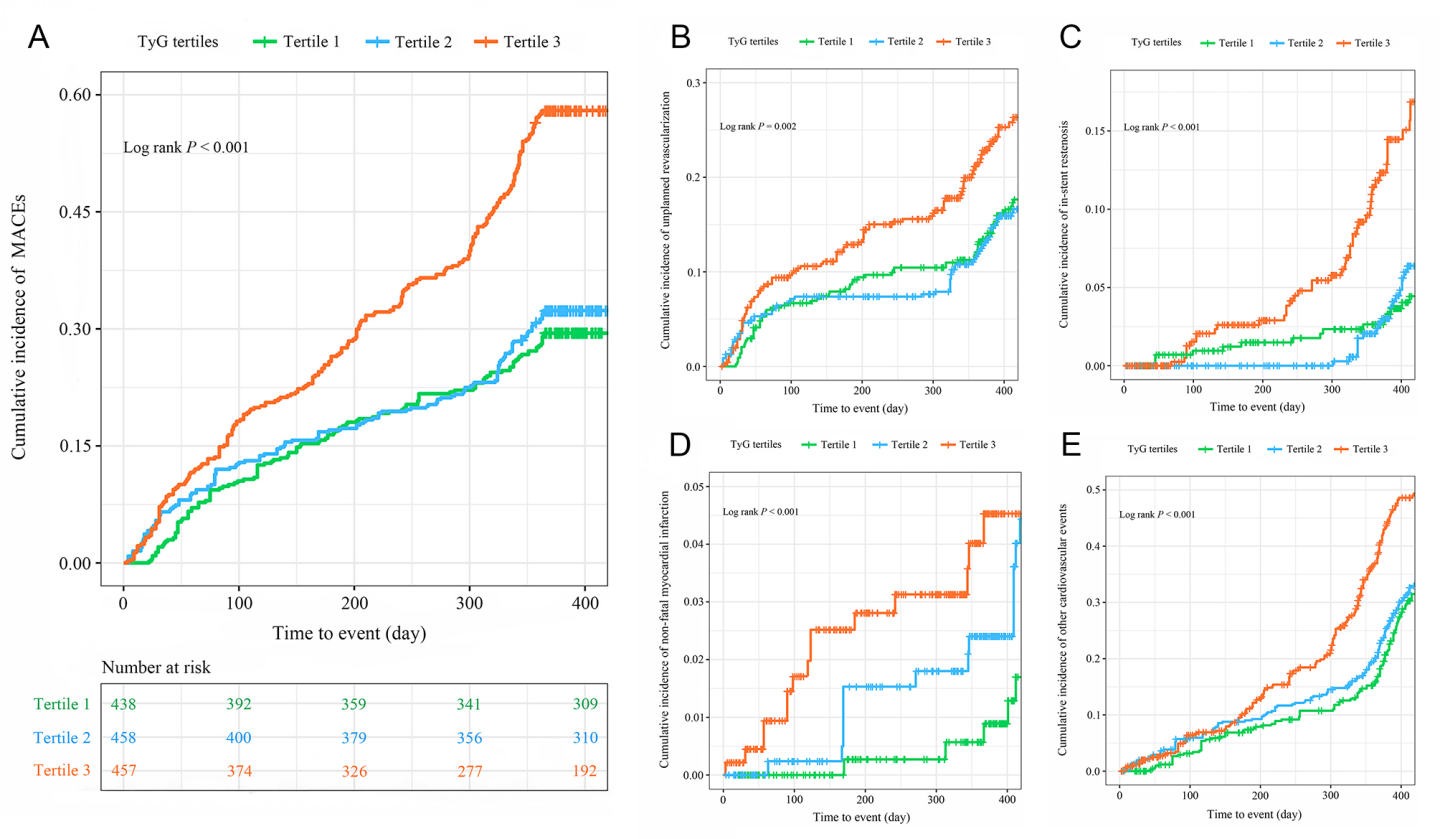
Kaplan-Meier curves for endpoints grouped by the TyG index tertiles. The cumulative incidence of **(A)** major adverse cardiovascular events (MACEs), (**B)** unplanned revascularization, (**C)** in-stent restenosis, (**D)** non-fatal myocardial infarction, and (**E)** other cardiovascular events during follow-up grouped according to the TyG index tertiles was analyzed by Kaplan-Meier analysis. The *P* value was calculated with the log-rank test. TyG, triglyceride-glucose; MACE, major adverse cardiovascular event 185B208

A multivariate RCS analysis was conducted to determine whether there was a potential linear or nonlinear association between the TyG index and the endpoints in CCS patients undergoing PCI. As can be seen in Fig. 3, We recorded that the TyG index was proved to have a nonlinear relationship with the probability of the risk of MACEs within one year according to the RCS mode (*P* for non-linearity < 0.001). Furthermore, we defined three categories of included patients based on the TyG index tertiles. The Kaplan-Meier analysis for endpoints grouped by the TyG index tertiles were shown in Fig. 4. The probability of cumulative incidences of MACEs such as unplanned revascularization, in-stent restenosis, non-fatal myocardial infarction and other cardiovascular events was significantly higher in patients with a higher TyG index than in those with a lower TyG index (all Log rank *P* < 0.05).

Table 2 describes the results of the multivariate Cox proportional hazards regression analysis, revealing an association between the TyG index score and an elevated risk of one-year endpoints (HR 1.595, 95% *CI* 1.370 ~ 1.855, *P* < 0.001). The unadjusted model 1 indicated that the TyG index was statistically significantly associated with one-year endpoints, and the T3 was at elevated risk for an endpoint event. After adjusting for age, gender, BMI, SBP, and DBP in model 2, the TyG index as a continuous variable was an independent predictor for the endpoints (HR 1.742, 95% *CI* 1.504 ~ 2.016, *P* < 0.001). After further adjusting for diabetes, hypertension, familial CVDs, smoking and drinking history, TC, LDL-C, HCY, Hs-CRP, number of coronary lesions and stent implantation in model 3, the TyG index still remained independently associated with one-year endpoints (*P* < 0.001). Moreover, taking the T1 in as a reference, the risks of the primary endpoint were 2.522-fold higher (HR 2.522, 95% *CI* 2.032 ~ 3.128, *P* < 0.001) and 2.297-fold higher (HR 2.297, 95% *CI* 1.842 ~ 2.864, *P* < 0.001) in the T3 of Model 2 and Model 3, respectively. The trend analyses from T1 to T3 for the three models were all statistically significant (all *P* for trend < 0.001).

**Table 2:** The association between various TyG index groups and endpoints

	Model 1		Model 2		Model 3	
	HR (95% *CI*)	*P* value	HR (95% *CI*)	*P* value	HR (95% *CI*)	*P* value
TyG index	1.587 (1.382 ~ 1.822)	< 0.001	1.742 (1.504 ~ 2.016)	< 0.001	1.595 (1.370 ~ 1.855)	< 0.001
TyG tertiles						
T1	1 (reference)		1 (reference)		1 (reference)	
T2	1.313 (0.985 ~ 1.749)	0.063	1.240 (0.976 ~ 1.573)	0.078	1.343 (1.054 ~ 1.711)	0.017
T3	3.339 (2.238 ~ 4.982)	< 0.001	2.522 (2.032 ~ 3.128)	< 0.001	2.297 (1.842 ~ 2.864)	< 0.001
*P* for trend		< 0.001		< 0.001		< 0.001
Model 1	Unadjusted					
Model 2	Adjusted for age, gender, BMI, SBP, and DBP					
Model 3	Adjusted for age, gender, BMI, SBP, DBP, diabetes, hypertension, familial CVDs, smoking and drinking history, TC, LDL-C, HCY, Hs-CRP, number of coronary lesions and stent implantation					

TyG, triglyceride-glucose; T1, tertile 1; T2, tertile 2; T3, tertile 3; HR, hazard ratio; CI, confidence interval; BMI, body mass index; SBP, systolic blood pressure; DBP, diastolic blood pressure; CVD, cardiovascular disease; TC, total cholesterol; LDL-C, low-density lipoprotein cholesterol; HCY, homocysteine; Hs-CRP, hypersensitive C-reactive protein

### Predictive ability test

**Fig. 5:**
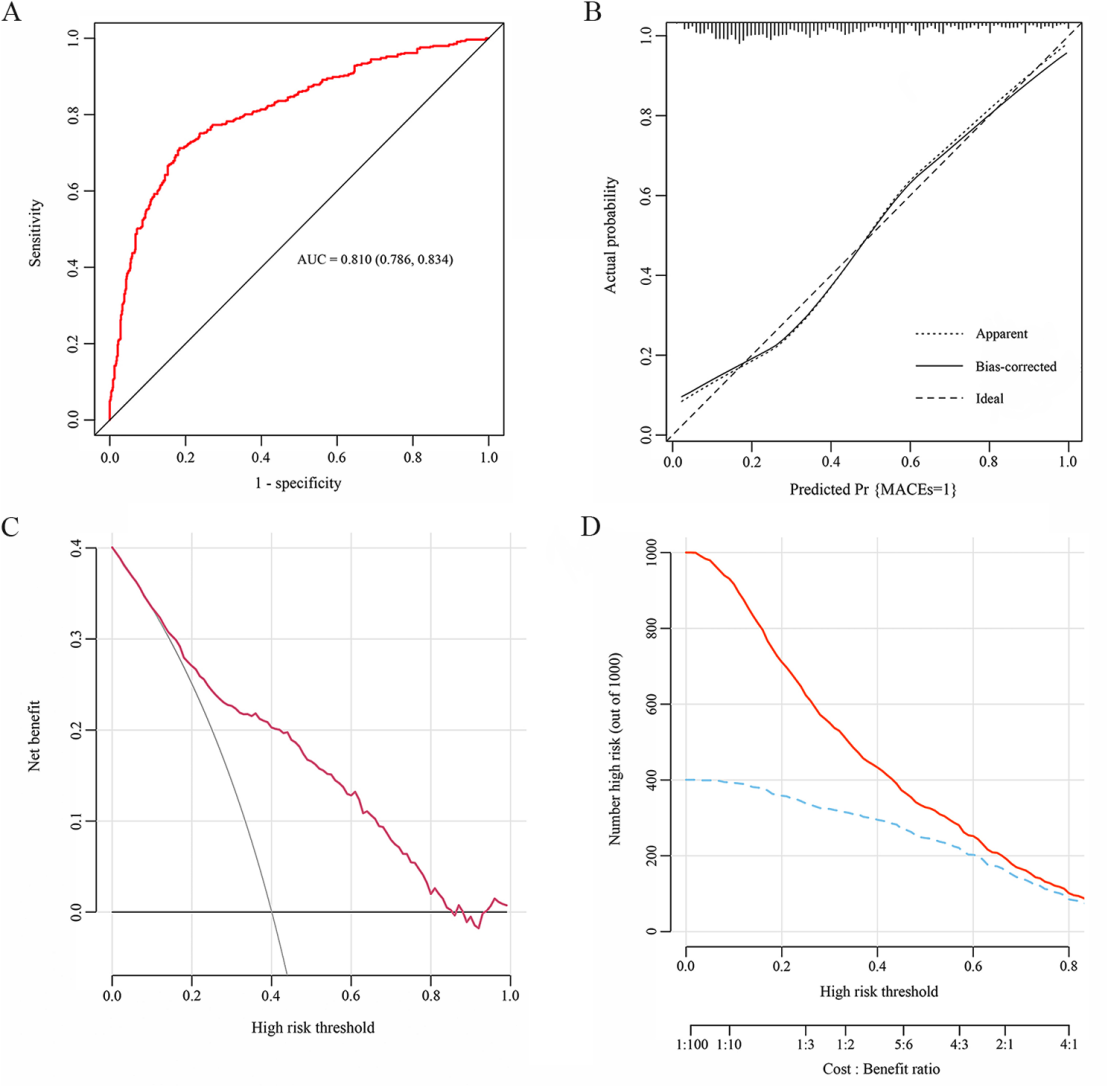
Performance evaluation of the TyG index for predicting endpoints. After adjustment for all confounders, predictive ability of the TyG index for endpoints was assessed using **(A)** the receiver operating characteristic (ROC) curve, (**B)** calibration curve, (**C)** decision curve analysis (DCA), and (**D)** clinical impact curve (CIC) analysis. AUC, the area under the ROC curve; MACE, major adverse cardiovascular event

The ROC curve, calibration curve, DCA, and CIC were performed to comprehensively identify the predictive power of the TyG index for endpoints (Fig. 5). After adjustment for all confounders, the ROC curve demonstrated a high predictive ability of the TyG index for endpoints with an AUC of 0.810 (0.786, 0.834) and the calibration curve indicated an excellent goodness-of-fit of the multivariate model using the *χ*^*2*^ likelihood ratio test (*χ*^*2*^ = 7.474, *P* = 0.486). In addition, DCA and CIC analysis were conducted to assess the clinical utility of the model, suggesting that the model had a good overall net benefit and clinical impact within most reasonable threshold probability.

### Subgroup analysis

To confirm the relationship between the TyG index and the risk of MACEs stratified by age, gender, BMI, diabetes, hypertension, familial CVDs, LDL-C, multi-vessel disease and multi-DES implantation, subgroup analyses were carried out. As shown in Table 3, the results of subgroup analyses were almost consistent with the major findings of the study. Patients in higher TyG index tertile group tended to have a higher incidence of MACEs within one year across all subgroups. Meanwhile, LDL-C level and the number multi-DES implantation were found to interact with the relationship between the TyG index and endpoints (*P* < 0.05).

**Table 3:** Cox proportional hazards analysis evaluating prognostic implication of TyG index in various subgroups

Variables	Case	TyG tertiles [HR (95% *CI*)]			*P* for trend	*P* for interaction
		T1	T2	T3		
Age (years)						
< 65	687	1 (reference)	1.306 (0.870 ~ 1.960)	2.647 (1.828 ~ 3.834)	< 0.001	0.747
≥ 65	666	1 (reference)	1.153 (0.859 ~ 1.549)	2.692 (2.074 ~ 3.496)	< 0.001	
Gender						
Male	979	1 (reference)	1.169 (0.895 ~ 1.527)	2.071 (1.622 ~ 2.643)	< 0.001	0.318
Female	374	1 (reference)	1.002 (0.606 ~ 1.658)	3.399 (2.214 ~ 5.218)	< 0.001	
BMI (kg/m^2^)						
< 24	482	1 (reference)	0.915 (0.570 ~ 1.469)	2.898 (1.926 ~ 4.361)	< 0.001	0.121
≥ 24	871	1 (reference)	1.162 (0.884 ~ 1.528)	2.028 (1.584 ~ 2.595)	< 0.001	
Diabetes						
Yes	612	1 (reference)	0.902 (0.619 ~ 1.315)	2.172 (1.571 ~ 3.002)	< 0.001	0.083
No	741	1 (reference)	1.274 (0.940 ~ 1.726)	2.345 (1.757 ~ 3.130)	< 0.001	
Hypertension						
Yes	1026	1 (reference)	1.236 (0.931 ~ 1.640)	2.610 (2.020 ~ 3.372)	< 0.001	0.573
No	327	1 (reference)	0.923 (0.589 ~ 1.447)	1.979 (1.346 ~ 2.910)	< 0.001	
Familial CVDs						
Yes	366	1 (reference)	1.267 (0.871 ~ 1.842)	1.893 (1.334 ~ 2.685)	< 0.001	0.982
No	987	1 (reference)	0.975 (0.717 ~ 1.325)	2.504 (1.922 ~ 3.261)	< 0.001	
LDL-C (mmol/L)						
< 1.8	299	1 (reference)	2.007 (1.303 ~ 3.092)	3.907 (2.501 ~ 6.102)	< 0.001	0.015
≥ 1.8	1054	1 (reference)	0.903 (0.681 ~ 1.197)	2.105 (1.649 ~ 2.686)	< 0.001	
Multi-vessel disease						
Yes	91	1 (reference)	1.497 (0.612 ~ 3.664)	3.509 (1.572 ~ 7.831)	0.004	0.701
No	1262	1 (reference)	1.092 (0.855 ~ 1.395)	2.296 (1.845 ~ 2.858)	< 0.001	
Multi-DES implantation						
Yes	302	1 (reference)	2.256 (1.456 ~ 3.496)	3.470 (2.356 ~ 5.111)	< 0.001	0.001
No	1051	1 (reference)	0.940 (0.710 ~ 1.245)	2.011 (1.559 ~ 2.593)	< 0.001	

TyG, triglyceride-glucose; T1, tertile 1; T2, tertile 2; T3, tertile 3; HR, hazard ratio; CI, confidence interval; BMI, body mass index; CVD, cardiovascular disease; LDL-C, low-density lipoprotein cholesterol; DES, drug-eluting stent

## Discussion

In our population-based study, we recorded an association between the TyG index and the risk of MACEs within one year in CCS patients undergoing PCI, and this relationship also remained significant even after adjusting for all confounding factors. Simultaneously, the results implied that a higher TyG index indicated a greater prevalence of MACEs within a certain range, and the highest TyG index values enhanced the risk by 59.5% over the 1-year follow-up in the population. Moreover, the TyG index had an excellent predictive performance according to the results of AUC and *χ*^*2*^ likelihood ratio test after adjustment for potential confounders. DCA and CIC analysis also revealed a good overall net benefit and clinical impact of the multivariate model. The multivariate RCS model showed that the TyG index had a nonlinear relationship with the probability of the risk of MACEs within one year. These findings revealed the prognostic value of the TyG index for MACEs in CCS patients undergoing PCI. Most importantly, this study suggests that a simple method of estimating IR may optimize the risk stratification of recurrent cardiovascular risk in CCS patients undergoing PCI.

IR is defined as a decrease in the efficiency of insulin in promoting glucose uptake and utilization, which reflects the disorder of the metabolic balance [23]. According to previous studies, IR is believed as an important risk factor for CVDs can lead to poor clinical outcomes in various ways, such as inducing endothelial dysfunction, causing systemic glucose-lipid metabolism disorders, and triggering oxidative stress and inflammatory response [24–27]. Conventional approaches for detecting IR mainly include the hyperinsulinemic-euglycemic clamp technique and the homeostasis model assessment for IR (HOMA-IR) [28]. Given the limitations of traditional assessment methods such as time-consuming nature, high cost and complexity, and instability of the results, it is difficult to apply them in practical clinical settings and large-scale studies.

The triglyceride-glucose (TyG) index was firstly proposed by Unger G et al. in 2013 as an alternative predictor of IR [29]. Recently, substantial studies have confirmed that the TyG index is not only strongly correlated with IR [30], even the risk of CVDs in general population [31], but also could be considered as a prognostic surrogate indicator of hypertension [32], heart failure [11], CAD [33], and other CVDs [34, 35]. Furthermore, the TyG index is strongly associated with HOMA-IR and HIEC, could be used to identify IR with high sensitivity (96.5%) and specificity (85.0%), even outperforming the HOMA-IR in evaluating IR [17, 36, 37]. At present, numerous clinical studies demonstrated that the TyG index has been considered as a comparatively extensive method in clinical research with regard to CVDs. Data from a study [38] of 30,291 subjects screened from the China National Diabetes and Metabolic Disorders Study proposed that the TyG index was simpler and more suitable for the identification of metabolically unhealthy individuals as well as who have high risk of cardiometabolic diseases among the Chinese adult population, comparing with other surrogate indices of IR. A study [39] investigated 5014 patients of the Vascular Metabolic CUN cohort with a 10-year follow-up and supported that the TyG index might be useful to early identify individuals at a high risk of developing CVDs, including coronary heart disease, cerebrovascular disease, and peripheral arterial disease. Besides, a significantly improvement of the predictive ability was recorded when the TyG index was added to the Framingham model, with AUCs ranging from 0.708 (0.68, 0.73) to 0.71 (0.70, 0.74) (*P* = 0.014). Similarly, two cohort studies [40, 41] showed that healthy participants with elevated TyG index may have a higher risk of cardiovascular events. Previous studies also confirmed that the TyG index was associated with cardiovascular risk factors and could be used as a useful predictive marker for CVDs both in diabetic [42] and non-diabetic population [43]. Further researches showed that the TyG index was substantially associated with arterial stiffness [44], coronary artery calcification [45], and carotid atherosclerosis [36], and could be regarded as a better predictor of cardiovascular risk than FPG or HbA1c in ACS patients undergoing PCI [46]. Among TG-derived metabolic indices such as the atherogenic index of plasma and TG to HDL-C ratio, the TyG index showed a better ability to predict the risk of MACEs in ACS patients [47–49].

In the current study, we found that a higher TyG index was associated with a greater prevalence of adverse cardiovascular events, which is consistent with the previous reports [39, 50]. Although some adverse cardiovascular events such as repeat revascularization was a controversial outcome in clinical trials for its subjective and biased nature, previous studies supported that it was significantly corrected with elevated risk for mortality and morbidity in the short term and composite safety events in the long term [51]. Meanwhile, the TyG index was determined positively associated with ischemia-driven revascularization and target vessel revascularization in ACS patients [52, 53]. In-stent restenosis is a delayed complication of stenting [54]. Observational studies discovered that patients with in-stent restenosis were more prone to develop ACS and adverse cardiovascular events at follow-up [55]. In addition, the TyG index was identified as an independent predictor of in-stent restenosis in ACS patients, indicating a prospect for the TyG index in in-stent restenosis assessment [56]. In this context, this study was designed to focus on the adverse cardiovascular events such as repeat revascularization and in-stent restenosis in the CCS population, which reflects not only the target lesion failure but also the progression of non-target lesions. Consistently, our study suggested that the TyG index was significantly associated with MACEs in a nonlinear relationship among CCS patients undergoing PCI after adjustment for all confounding factors and the highest TyG index values enhanced the risk by 59.5% over the 1-year follow-up in the population. Consequently, the present study powered by adverse cardiovascular events among the CCS population has extended the association between the TyG index and CAD, indicating that the TyG index could serve as a potent prognostic indicator for risk stratification in CCS patients undergoing DES-PCI.

In the analysis of the association between the TyG index and endpoints, we used three unadjusted and adjusted models to develop the multivariate Cox proportional hazards regression analysis. Model 2 only adjusted for demographics (age, gender, and BMI) and blood pressure, which can be ascertained easily, showed great usefulness and generality in clinical practice. While the almost fully adjusted model (Model 3) provided the most specific risk prediction model and showed the independent prediction value of the TyG index for the incidence of MACEs within one year which cannot be explained by other covariates. Meanwhile, consistent with the major findings of the study, the results of subgroup analyses implied that age, gender, BMI, diabetes, hypertension, familial CVDs, LDL-C, multi-vessel disease and multi-DES implantation were the crucial risk factors of adverse cardiovascular events in CCS patients undergoing PCI. Additionally, our findings indicated that patients with higher baseline TyG index were more likely to have altered cardiac structure and worse peripheral artery conditions, which was manifested by higher LAD, LVDd, IVST, PWT and lower FMD, ABI, respectively. In general, the innovative finding of this study was that the cardiac structure and peripheral artery function were valuable factors for cardiovascular prognosis. Furthermore, population-based studies indicated that the TyG index be identified to be independently associated with aortic intima-media thickness [57], left ventricular functional impairment and structure abnormality [58], and cardiac hemodynamics [59], and an increased risk of incident PAD [60].

Nevertheless, some limitations must be acknowledged. Firstly, this was a single-center, retrospective study based on Chinese patients, selection bias may be introduced and the generalizability of our results need to be further demonstrated externally. Secondly, only baseline measurements at admission were available, and data collected at different time points during the follow-up period were lacking, which may lead to deviations in the analysis results. Thirdly, although some confounders were adjusted, our research results would still be affected by residual confounding factors. In addition, due to the limited clinical information collected, the differences between the TyG index and other predictors for the prognosis of CCS patients undergoing PCI still need to be investigated. Thus, further prospective, multicenter studies with larger sample sizes and multi-time node information need to be conducted to make our findings more reliable.

## Conclusion

Overall, our research indicated that the TyG index could be considered as a prognostic indicator of adverse cardiovascular events such as unplanned revascularization, in-stent restenosis, non-fatal myocardial infarction and other cardiovascular outcomes in CCS patients undergoing PCI. In the high-risk group, TyG might be a valuable tool for risk categorization and management. Further studies are needed to confirm our findings and examine the potential mechanisms between TyG and the population.

### Electronic supplementary material

Below is the link to the electronic supplementary material.

Supplementary Material 1

## Data Availability

The data of the study population were extracted from the Hospital Information System. The datasets are not publicly available because the individual privacy of the participants should be protected. Data are however available from the corresponding author on reasonable request.

## Abbreviations

TyG: Triglyceride-glucose
CCS: Chronic coronary syndrome
PCI: Percutaneous coronary intervention
MACE: Major adverse cardiovascular event
CAD: Coronary artery disease
CVD: Cardiovascular disease
DES: Drug-eluting stent
ACS: Acute coronary syndrome
CABG: Coronary artery bypass grafting
IR: Insulin resistance
RCS: Restricted cubic spline
ROC: Receiver operating characteristic curve
AUC: Area under curve
DCA: Decision curve analysis
CIC: Clinical impact curve
BMI: Body mass index
OMI: Old myocardial infarction
ACEI: Angiotensin converting enzyme inhibitor
ARB: Angiotensin receptor blocker
Neu: Neutrophil
Lym: Lymphocyte
PLT: Platelets
Hb: Hemoglobin
ALT: Alanine aminotransferase
AST: Aspartate aminotransferase
TC: Total cholesterol
TG: Triglyceride
LDL-C: Low-density lipoprotein cholesterol
HDL-C: High-density lipoprotein cholesterol
Lp (a): Lipoprotein (a)
HCY: Homocysteine
Hs-CRP: Hypersensitive C-reactive protein
Scr: Serum creatinine
FBG: Fasting blood glucose
HbA1c: Glycosylated hemoglobin
PAD: Peripheral artery disease
baPWV: Brachial-ankle pulse wave velocity
ABI: Ankle-brachial index
FMD: Brachial artery flow-mediated vasodilatation
LAD: Left atrial diameter
LVEF: Left ventricular ejection fraction
LVDd: Left ventricular end-diastolic diameter
IVST: Interventricular septal thickness
PWT: Left ventricular posterior wall thickness
