# Supplementary material for: Prognostic value of triglyceride-glucose index in patients with chronic coronary syndrome undergoing percutaneous coronary intervention
Source: Cardiovasc Diabetol. 2023 Nov 28;22:322. doi: 10.1186/s12933-023-02060-7 (PMC10685592; doi:10.1186/s12933-023-02060-7)
Supplement: Supplementary file 1 — Supplementary Material 1 [file 12933_2023_2060_MOESM1_ESM.pdf]

Additional file 1

Table S1 Baseline characteristics of the study population according to the endpoints

Variables	MACEs (*n*=542)	Non-MACEs (*n*=811)	*P* value
TyG index	9.04 (8.62, 9.28)	8.72 (8.42, 9.02)	<0.001
Demographics			
Male (n, %)	396 (73.06)	583 (71.89)	0.635
Age (years)	67 (61, 76)	62 (55, 69)	<0.001
BMI (kg/m^2)	25.7 (24.13, 27.77)	24.39 (22.86, 26.73)	<0.001
SBP (mmHg)	137 (122, 149)	134 (125, 140)	0.003
DBP (mmHg)	79 (70, 85)	78 (74, 83)	0.892
HR (bpm)	75 (70, 82)	74 (68, 75)	<0.001
Case history (n, (%)			
Smoking	249 (45.94)	350 (43.16)	0.312
Drinking	143 (26.38)	157 (19.36)	0.002
Diabetes	267 (49.26)	345 (42.54)	0.015
Hypertension	405 (74.72)	621 (76.57)	0.436
Stroke	113 (20.85)	120 (14.8)	0.004
OMI	153 (28.23)	85 (10.48)	<0.001
Dyslipidemia	425 (78.41)	464 (57.21)	<0.001
Familial CVDs	215 (39.67)	151 (18.62)	<0.001
Coronary lesions (n, %)			
One-vessel disease	377 (69.56)	586 (72.26)	0.283
Two-vessel disease	120 (22.14)	179 (22.07)	0.976
Multi-vessel disease	45 (8.3)	46 (5.67)	0.058
Number of stents (n, %)			
One-DES implantation	189 (34.87)	436 (53.76)	<0.001
Two-DES implantation	168 (31)	258 (31.81)	0.751
Multi-DES implantation	185 (34.13)	117 (14.43)	<0.001
Cardiovascular medications (n, %)			
Aspirin	529 (97.6)	808 (99.63)	0.001
Clopidogrel/Ticagrelor	539 (99.45)	809 (99.75)	0.362
Statins	527 (97.23)	778 (95.93)	0.205
ACEI/ARB	287 (52.95)	384 (47.35)	0.043
β-blockers	409 (75.46)	541 (66.71)	0.001
CCB	164 (30.26)	276 (34.03)	0.146
Nitrates	146 (26.94)	215 (26.51)	0.862
Diuretics	152 (28.04)	126 (15.54)	<0.001
Laboratory measurements			
Neu (10^9/L)	4.36 (3.36, 5.42)	4.36 (3.4, 4.66)	0.044
Lym (10^9/L)	1.63 (1.29, 2.02)	1.81 (1.54, 2.14)	<0.001
PLT (10^9/L)	205 (168, 246)	209 (184, 235)	0.043
Hb (g/L)	134 (120, 144)	134 (130, 145)	<0.001
ALT (U/L)	20 (13, 32)	21 (15, 30)	0.017
AST (U/L)	19 (15, 26)	19 (16, 24)	0.441
TC (mmol/L)	3.92 (3.27, 5.09)	3.81 (3.18, 4.54)	0.001
TG (mmol/L)	1.63 (1.13, 2.02)	1.28 (1.02, 1.75)	<0.001
LDL-C (mmol/L)	2.35 (1.85, 3.13)	2.27 (1.86, 2.83)	0.014
HDL-C (mmol/L)	1 (0.82, 1.15)	1.01 (0.88, 1.18)	0.014
Lp (a) (mg/L)	170.14 (63.57, 309.5)	150.72 (51.76, 213.47)	<0.001
HCY (μmol/L)	15.65 (12.21, 20.38)	12.88 (10.87, 16.11)	<0.001
Hs-CRP (mg/L)	3.59 (1.56, 6.69)	1.58 (0.71, 3.78)	<0.001
Scr (μmol/L)	75.5 (63.5, 89.5)	73.2 (61.6, 83.4)	0.001
FBG (mmol/L)	6.17 (5.22, 7.18)	6.1 (5.21, 6.26)	<0.001
HbA1c (%)	6.4 (5.8, 7.5)	6 (5.6, 6.7)	<0.001
PAD indicators			

baPWV (m/s)	21.91 (19.91, 24.21)	15.68 (14.72, 16.82)	<0.001
ABI	0.94 (0.81, 1.01)	1.16 (1.1, 1.2)	<0.001
FMD (%)	6.1 (5.6, 6.43)	7.9 (7, 9.2)	<0.001
Echocardiography			
LAD (mm)	39 (36, 42)	36 (35, 38)	<0.001
LVEF (%)	62 (55, 67)	67 (63, 71)	<0.001
LVDd (mm)	53 (50, 56)	49 (46, 51)	<0.001
IVST (mm)	11 (10, 12)	10 (9, 10)	<0.001
PWT (mm)	10 (9, 10)	9 (8, 9)	<0.001

TyG, triglyceride-glucose; MACE, major adverse cardiovascular event; BMI, body mass index; SBP, systolic blood pressure; DBP, diastolic blood pressure; HR, heart rate; OMI, old myocardial infarction; CVD, cardiovascular disease; DES, drug-eluting stent; ACEI, angiotensin converting enzyme inhibitor; ARB, angiotensin receptor blocker; CCB, calcium channel blockers; Neu, neutrophil; Lym, lymphocyte; PLT, platelets; Hb, hemoglobin; ALT, alanine aminotransferase; AST, aspartate aminotransferase; TC, total cholesterol; TG, triglyceride; LDL-C, low-density lipoprotein cholesterol; HDL-C, high-density lipoprotein cholesterol; Lp (a), lipoprotein (a); HCY, homocysteine; Hs-CRP, hypersensitive C-reactive protein; Scr, serum creatinine; FBG, fasting blood glucose; HbA1c, glycosylated hemoglobin; PAD, peripheral artery disease; baPWV, brachial-ankle pulse wave velocity; ABI, ankle-brachial index; FMD, brachial artery flow-mediated vasodilatation; LAD, left atrial diameter; LVEF, left ventricular ejection fraction; LVDd, left ventricular end-diastolic diameter; IVST, interventricular septal thickness; PWT, left ventricular posterior wall thickness

Table S2 Collinearity diagnostics among the variables

Variables	VIF
Demographics	
Gender	2.016
Age	1.804
BMI	1.336
SBP	1.699
DBP	1.889
HR	1.258
Case history	
Smoking	1.716
Drinking	1.423
Diabetes	1.684
Hypertension	1.602
Stroke	1.108
OMI	1.185
Dyslipidemia	1.259
Familial CVDs	1.110
Coronary lesions	1.743
Number of stents	1.722
Cardiovascular medications	
Aspirin	1.165
Clopidogrel/Ticagrelor	1.045
Statins	1.073
ACEI/ARB	1.335
β-blockers	1.110
CCB	1.257
Nitrates	1.119
Diuretics	1.225
Laboratory measurements	
Neu	1.325
Lym	1.260
PLT	1.254
Hb	1.748
ALT	1.597
AST	1.706
TC	1.841
TG	5.628
LDL-C	1.781
HDL-C	1.117
Lp (a)	1.105
HCY	1.191
Hs-CRP	1.284
Scr	1.358
FBG	2.902
HbA1c	2.226
TyG index	7.783
PAD indicators	
baPWV	2.265
ABI	2.185
FMD	1.511
Echocardiography	
LAD	1.739
LVEF	1.761
LVDd	2.145
IVST	1.857

PWT	1.772

VIF, variance inflation factor; BMI, body mass index; SBP, systolic blood pressure; DBP, diastolic blood pressure; HR, heart rate; OMI, old myocardial infarction; CVD, cardiovascular disease; DES, drug-eluting stent; ACEI, angiotensin converting enzyme inhibitor; ARB, angiotensin receptor blocker; CCB, calcium channel blockers; Neu, neutrophil; Lym, lymphocyte; PLT, platelets; Hb, hemoglobin; ALT, alanine aminotransferase; AST, aspartate aminotransferase; TC, total cholesterol; TG, triglyceride; LDL-C, low-density lipoprotein cholesterol; HDL-C, high-density lipoprotein cholesterol; Lp (a), lipoprotein (a); HCY, homocysteine; Hs-CRP, hypersensitive C-reactive protein; Scr, serum creatinine; FBG, fasting blood glucose; HbA1c, glycosylated hemoglobin; TyG, triglyceride-glucose; PAD, peripheral artery disease; baPWV, brachial-ankle pulse wave velocity; ABI, ankle-brachial index; FMD, brachial artery flow-mediated vasodilatation; LAD, left atrial diameter; LVEF, left ventricular ejection fraction; LVDd, left ventricular end-diastolic diameter; IVST, interventricular septal thickness; PWT, left ventricular posterior wall thickness

No potentially significant collinearity is defined as VIF less than 10 among variables
